# Chronic pain as a human rights issue: setting an agenda for preventative action

**DOI:** 10.1080/16549716.2017.1348691

**Published:** 2017-08-02

**Authors:** Louise Frenkel, Leslie Swartz

**Affiliations:** ^a^ Department of Psychiatry and Mental Health, University of Cape Town, Cape Town, South Africa; ^b^ Department of Psychology, Stellenbosch University, Stellenbosch, South Africa

**Keywords:** chronic pain, human rights, post-traumatic stress disorder, continuous traumatic stress

## Abstract

**Background**: Historically, chronic pain has been viewed primarily as a medical issue, and research has been focused on the individual and predominantly on pain sufferers in high-income countries.

**Objectives**: This article argues the need for a broader understanding of the context of chronic pain and its complex aetiologies and maintenance. It is suggested that the interaction between chronic pain and social context has been inadequately explored.

**Methods**: A single case study is used of a man living in a violent urban environment in South Africa accessing a pain clinic at a tertiary hospital. Following the case-study approach, as used in the chronic traumatic stress field by Kaminer et al., the case material is utilised to develop an argument for a new research agenda.

**Results**: Analysis of the case material demonstrates the complex interplay between bodily and psychological experiences, with chronic pain being contextually maintained and exacerbated by very difficult life circumstances, ongoing violence, and marginalisation.

**Conclusions**: It is suggested that a research agenda be developed which explores the links between chronic pain and ongoing chronic traumatisation in contexts of continuous violence, oppression, and disempowerment – common features of much of the contemporary majority world.

## Background

Chronic pain is a major global health issue. The International Association for the Study of Pain (IASP) Task Force for the Classification of Chronic Pain estimates that it affects 20% of people worldwide, and accounts for 15–20% of physician visits [1]. Because of its enduring nature, chronic pain is often experienced as disabling, affecting people’s mobility, ability to work, social relationships, and, in many cases, their psychological sense of themselves and their place in the world [2].

Historically, chronic pain has been defined primarily as a medical issue affecting individuals (rather than communities), which has meant that much of the research has focused on symptoms, diagnosis, individual vulnerability, and the immediate relationship context [3]. It is also the case that most of what is known about chronic pain comes from research conducted in high-income countries, where health-care resources are plentiful compared to most countries in the world and where social and political conditions are relatively stable. If globally relevant approaches to dealing with chronic pain are to be developed, however, it is important to have a broader contextual view of chronic pain, its complex aetiologies, and its maintenance. In terms of the right to health, it is essential that this complex phenomenon is understood in broader global contexts [4,5]. The precise nature of the relationship between chronic pain and social context remains relatively unexplored at this stage. In terms of the relationship between chronic pain and post-traumatic stress disorder (PTSD), a number of pathways of the relationship have been suggested, many focussing on the question of sensitisation and inflammation in bodily systems following traumatic events [6–8]. There is, however, an emerging literature which argues that the PTSD model does not adequately capture the full complexity of the experience of ongoing chronic traumatic stress in highly unstable environments – environments in which many of the world’s population live [9,10]. It is important to begin to build an evidence base for understanding the relationships between pain and environment in these unstable contexts, and as a first step towards this, the detailed use of case material may suggest directions for further research and action.

With these considerations in mind, therefore, this study used a single case study to suggest developing a research agenda which explores the links between chronic pain and ongoing chronic traumatisation in contexts of continuous violence, oppression, and disempowerment – common features of much of the contemporary majority world.

The article begins with a case study of a presentation of chronic pain at a South African hospital. This leads to a discussion of the links between chronic pain and PTSD. Following this is a discussion of how contemporary thinking about chronic stress syndromes may expand and deepen the understanding of chronic pain globally and how this might set the agenda for further research.

## Methods

The case-study method has been extensively used in health research, as it is especially useful for explicating health issues which have not been studied extensively [10,11]. Case studies are particularly helpful in giving rich information about issues which may be used for future research and for setting agendas for future, larger-scale studies [12,13]. For the current research, the case-study approach outlined by Kaminer et al. [14] in their work on continuous traumatic stress in South Africa was followed. A key feature of their approach to case studies is the careful attention not only to the personal context of health issues but also to the political context. It is also essential in case studies on health issues which comment on health-system organisational factors to attend closely to the health-system context in which illness is experienced and treated [11,13,15]. Stake [16] has produced a detailed checklist on issues of quality in case-study research, and this checklist was followed in writing the present case study.

The analytic steps used in the analysis of the case study move from the more narrowly clinical towards the more contextual, following the approach within this field, as adopted by Kaminer et al. [14] and in keeping with the role of this case study in an interpretive context [15,16].

As a case study does not use sampling, the aim is not to generalise to the population as a whole, as in quantitative studies which use inferential statistics for this purpose [17]. The aim, by contrast, is to generate hypotheses and issues for consideration in future research [11]. This method is therefore especially appropriate for formative work such as the present study, which leads to agenda-setting for future work.

As Scholz and Tietje [15] note, the key question in selecting a case study (or, indeed, for using a case study as part of a scholarly argument) rests with the purpose of the use of the case. In the current study, the purpose of the case, in what is hoped is a newly developing field, is to elucidate as clearly as possible key aspects of the complex interplay between ongoing social factors and the experience of chronic pain. The decision was therefore made to present a case that was well known to the authors and familiar to the clinical context within which the first author works.

The first author (LF) works as a clinical psychologist in a state hospital in a South African city. Part of her work is in the Pain Clinic – an outpatient service of this tertiary referral general hospital. The state hospital sector caters largely for patients from areas characterised by poverty and unemployment, and commonly where there are high levels of violence of various kinds, including gender-based violence and gang violence.

Contextual issues affecting patients came starkly into focus when LF began treating a patient (‘R’), who presented with complex regional pain syndrome (CRPS) and PTSD, living in a community in which gang warfare is rife. He had been caught in the crossfire between two rival gangs as he was exiting a taxi, and he sustained a gunshot wound to his lower left leg, which developed into CRPS [18]. The vast majority of patients experiencing chronic pain in this city (and throughout the country) will not be referred to a service such as this. Because this patient had been previously treated in the hospital, however, he was referred to the hospital-based service and remained as a patient on an ongoing basis. Chronic-pain services are not well developed at the primary-care level – hence he was followed up within a tertiary hospital outpatient setting.

In terms of ethical considerations, informed consent was obtained from the patient in accordance with formal ethical requirements. It is also important in case-study research in particular, where some detail is presented, to consider the question of whether a case is so unusual that the participant will be easily identifiable and confidentiality broken. Some minor details have therefore been changed (and others not reported) for ethical reasons. Furthermore, the choice of this particular case as exemplary of broader social conditions makes it extremely unlikely that the particular patient would be identified. In this regard, and to safeguard the patient further, the draft of this article was shown to a number of clinical colleagues, and all commented that the case and experience of pain in the context of ongoing marginalisation and violence is familiar and common territory for those dealing with cases of this nature, and not unique to a particular case.

## Results

### Chronic pain, marginalisation, and violence: a case study

#### Participant characteristics

R is a 41-year-old man living in a small house with his wife, five children, and extended family, on the boundary of the territory of two rival gangs. Before his injury (in 2012), he worked as a builder, but he has been unemployed since the incident. He had to leave school in grade 7 in order to work, and has had no opportunity for further training. Currently, he receives a Disability Grant from the government, and he and his family survive on this, along with some income from his wife who sells vegetables from their home. His daily life is rather restricted. He spends his time at home mostly isolated and idle, and does a small amount of childcare in his small cramped home.

#### Case description

##### Diagnosis of chronic pain

After the shooting, R was brought to the hospital, given medical treatment, and, after a few days, sent home. He received ongoing outpatient treatment (physiotherapy and medication), but he continued to complain of pain in his leg and ankle, and he was referred to the Pain Clinic seven months after the injury. He was assessed as having possible CRPS and a depressed mood. CRPS is a pain condition which usually follows an injury; occurs regionally, usually in limb extremities; is associated with oedema, abnormal skin blood flow, colour, and temperature changes; changes in hair growth; and limitations in movement [18]. He was prescribed the appropriate medication (painkillers and an antidepressant to help with mood and sleep), and referred to the physiotherapist for specialist ‘mirror therapy’ and the psychologist on the multidisciplinary team.

##### Psychological treatment

Psychological treatment at the pain clinic generally involves short-term interventions (four to six sessions), with one or two follow-up sessions coinciding with visits to the clinic to collect medication. This has not been the case for R. After the initial intervention, R has continued to attend the psychology sessions at the pain clinic on a monthly and then three-monthly basis for the last two-and-a-half years. The reasons for this are complex but raise the important question of the impact of R’s experience and social context on the intransigence of his chronic pain and PTSD symptoms.

Listening to R’s story about the shooting, but also about his current life situation and his daily routine, it became clear that it was not only the pain that was limiting his functioning, but also the continuing impact of the trauma on his sense of safety, primarily in his home and neighbourhood, manifesting in symptoms of PTSD.

A diagnosis of PTSD involves four distinct symptom clusters: re-experiencing the trauma; avoiding any reminder of the trauma; negative cognitions and mood; and hyperarousal (DSM, 5th ed.) [19,20]. All of these are evident in R’s accounts of his daily life.

##### Symptoms of living with trauma and ways of coping

R describes vivid flashbacks of the shooting, brought on when he hears gunfire (which is almost daily), but which can also be triggered by any loud noise. His response is to hide in his bedroom, and take some of whatever medication he has on hand, which sedates him and allows him to sleep. His anxiety is acute but is not a full panic attack. He also speaks of vivid nightmares in which he has been shot and is trapped and helpless.

Retreating to his bedroom and sedating himself is a way of avoiding what he perceives to be an imminent threat. This avoidance generalises to a need to avoid any future potential danger, and keeps him in the house, sleeping or watching TV. He cannot easily avoid the place he was shot, as it is the taxi rank near his house, but he avoids being out on the street as much as possible. His avoidance keeps him at home but does not extend to agoraphobia, as he is able to attend his sessions at the pain clinic (he is always anxious to have an early appointment and to leave before it gets dark). Besides these appointments, and occasional visits to his sister who lives a few blocks away from him, he seldom ventures out of the house.

R presents with a depressed mood, a negative view of himself and his future, and a marked lack of interest or pleasure in almost all activities ‘most of the day, nearly every day’ [19]. He also has general fatigue (which may be due to his irregular self-medicating), feelings of worthlessness, and problems with thinking and concentration. He often speaks about death and has had chronic suicidal ideation since his mother’s death 10 years ago.

He says: ‘I’ve got so many things going through my head … voices telling me to hang myself … worrying about my children, what’s going to happen to them…’. However, he struggles to expand on these thoughts or discuss his concerns in any detail. It may be that it is not so much the content of each thought that is important but that he is trying to convey his experience of being in a constant state of anxiety and anger, his head filled with negative thoughts about the world and himself. He is trapped in his head in a persecutory world.

His thoughts about the trauma range from seeing it as a punishment for ‘some bad things I’ve done in my life’ to intense rage at the perpetrators and fantasies of revenge (which he realises, if acted on, would only serve to increase the real threat of danger). At the same time, he feels despondent and believes his life is over.

R manifests both the symptoms of major depressive disorder and the negative cognitions associated with PTSD, these diagnoses not being easily distinguishable. One of the emotional and behavioural symptoms that accompany PTSD is the expression of the fight (in fight or flight) response, in the form of irritable and angry outbursts that may be expressed as aggressive behaviour [19]. R is moody, irritable, and becomes angry and aggressive, to the extent that his family prefer him to retreat to the bedroom, and many of his friends no longer visit him. He has become socially isolated and prefers to be alone.

R’s angry outbursts can be seen as part of his state of arousal and heightened reactivity. When, due to his own fears, he shouts at his children to come inside and to lie on the floor, he frightens them and has at times hit them in his panic and rage. Often, he wakes up feeling angry and extremely frustrated that he cannot just ‘get up and walk … I just want to walk…’, and then the anger lasts all day. However, sometimes the anger is without reason, with no identifiable triggers. When it comes over him, he feels he is in a ‘different world’ and that the anger is controlling him. He then lashes out, mostly verbally, but also sometimes physically. He has never hurt his immediate family, but has had physical altercations with a member of his extended family and one or two friends. After one such outburst, the person had to be taken to hospital for suturing.

##### Symptoms in community and biographical context

The issue of aggressive behaviour is complicated in R’s case, as he both witnessed and was the victim of physical and verbal aggression as a child at the hands of his father. His mother was the primary target of his father’s aggression, but he was often caught in the violence between his parents. Some of R’s anger and frustration became a behavioural problem; he related the story of stabbing a boy at school with a pair of scissors. He acknowledges that aggression and violence are the only way he knows how to deal with difficult feelings and is afraid that his anger may lead him to hurt someone.

The concept of ‘Biographical Disruption’ [21] is a much cited concept in the literature on chronic illness, and it is helpful here in understanding R’s response. Bury [21] describes how a diagnosis of an acute or chronic illness (or arguably an experience of a traumatic injury) could change a person’s perception of themselves and of their future, making a clear point of ‘before’ and ‘after’ disruption in the trajectory of their lives. This disruption can involve a change in the person’s sense of themselves (their ‘identity’), their assumptions about their body, and their thoughts and expectations for the future. R believes that he is ‘useless’, that he will never work again, and that he has no future. His only reason for staying alive is the concern for his children. He believes nothing can change for him in the future: ‘When you’re shot, it’s like a lifetime of pain’.

Since his trauma, he has been unable to work, conduct normal social relationships, or function in any normal way. His struggle to re-engage in his life seems to be predominantly related to his state of mind rather than his injury, and suggests the significance of additional factors, particularly the co-occurrence of PTSD in this conundrum.

R has both PTSD and chronic pain, brought on by a traumatic injury. It could be argued that he would be predisposed to develop both syndromes because the trauma and injury coincided, but also possibly because of his early experience of trauma (his father’s abuse), which would make him more vulnerable to anxiety and would increase his physiological and emotional response to trauma (increased sensitisation) [22].

##### Sensitisation and embodied trauma

Sensitisation [22] renders him more vulnerable to triggers in the environment which leads to avoidant behaviours as a form of protection. R’s behaviour (avoiding movement, staying home, excessive sleeping, social withdrawal, etc.) is based on his fear of causing more physical pain, but even more so, it serves to protect him from exposure to any triggers that could re-evoke the trauma. He is keeping himself ‘safe’ in an ‘unsafe’ environment and protecting himself from any kind of stressful issue or task.

Pain becomes a coping behaviour – a form of embodied trauma – which is a powerful unconscious mechanism for keeping him isolated and disengaged, and ‘safe’ from the risk of repeated trauma. Miller [23] believed that the constant reviving of memories of a painful injury can ‘act as both a trigger and a re-enforcer of *chronic pain as a coping response to trauma*’ (p. 30, italics added).

The aim of treatment for R’s pain would be to help him to acknowledge the limitations it causes him and to find ways to work around these and to adjust and re-engage in his life as it is now. The avoidant behaviour which is part of PTSD, however, makes this extremely difficult. Addressing all the forms of avoidant behaviour is of course possible, but as described earlier, this has not helped to improve R’s quality of life.

So far, the focus has been on R’s response to trauma, but to what extent is the broader context of ongoing gang violence impacting on his individual response? Given this context, is it appropriate to be dismantling R’s avoidant behaviour?

R feels that his environment is dangerous. Regularly, he would begin his session by saying that the pain is ‘the same’ or ‘worse’ and then relate a story of yet another person or people who had been killed by the gangsters since his last visit to the hospital. On one occasion, he described hearing gunfire and discovering two of his neighbours shot dead, their bodies lying in the street. On another occasion he related the story of a neighbour who was wanted by the gangs but could not be found, so the gang had found his 12-year-old cousin and killed him as a warning. R knew this 12-year-old, who was often in his house with his own children.

R’s situation is complex. Not only is the sound of gunfire triggering (and amplifying) his pain and re-evoking his past trauma (in the present), but he is also re-experiencing acute trauma in the present, and is at continued risk of this being repeated. It is safe to say that he is living in a constant state of hyperarousal and an exacerbated sense of alertness to danger.

Clearly, R’s ongoing situation of life in a very violent context requires us to think more broadly than the co-morbidities between chronic pain and PTSD. The concept of PTSD is premised on the idea of an ‘ordinary’ (implicitly peaceful) life disrupted by an unusually stressful event, but R lives his life, as do many others all around the world, in a context in which violence and threat to existence are the norm.

## Discussions

It is important to emphasise that it is not the intention to generalise from a single case to an entire population – the present case is used to develop a more general argument for setting a future agenda. It is important to acknowledge that the presentation of the case is refracted through the clinical experience of the first author. While this may be seen as a strength in terms of familiarity with this kind of work, from a research point of view, the data cannot be viewed as objective and unaffected by previous clinical experience.

### PTSD, chronic pain, and continuous stress syndrome

When PTSD was first included in the psychiatric nomenclature in 1980 (DSM, 3rd ed.) [19], it referred to a set of anxiety symptoms related to the struggle to process a trauma that had already happened (thus ‘post’ in post-traumatic stress) but for which the after effects were being felt in the present, and it primarily applied to people’s response to war trauma or natural disasters.

Stevens et al. [24] trace the history of the development of PTSD, illustrating how historically it has dominated the research on trauma, reflecting the experience of high-income countries, to the exclusion of other forms of trauma which may be part of life for people in low-income countries, countries which are in the midst of civil conflict, or societies in which there is ongoing poverty, community violence, scarcity of resources, or political oppression [14].

Working in South Africa during the last stages of apartheid, Straker et al. [25] were the first to think about the impact of living in a situation of ongoing trauma, and although there has not been extensive exploration of the concept [26], there is an emerging literature looking at continuous stress in other countries [26–28].

Eagle and Kaminer [26] describe four ways in which continuous stress syndrome is different from PTSD: (1) the dangerous environment, (2) the fact that trauma is current and anticipated (3) that discriminating between real and perceived danger is complex, and (4) the absence of external protective systems (p. 89).

R’s life context meets all four of these criteria. He lives in a dangerous area in which there is the constant threat of violence. Neither he nor anyone else is able to predict when violent outbursts will occur. There are no neighbourhood resources to protect him or others now or in the foreseeable future.

The reality for R is that he has PTSD and chronic pain, in a social context which engenders continuous stress syndrome. He is trapped in a vicious cycle which makes managing his pain almost impossible. From a public-health and human-rights perspective, what is at stake for R (and for many people in similar circumstances) is that contextual factors – factors which health or psychological interventions as commonly understood cannot ameliorate – loom very large in the maintenance of ongoing symptoms, including the experience of physical pain [29].

### Chronic pain, human rights, and public health

As R’s story makes clear, chronic pain is a public-health and human-rights issue [30]. It is not new for chronic pain to be seen as a public-health issue, but the contextual factors discussed here – factors which are very common in the majority world – realign the argument. Traditionally, arguments about chronic pain as a public-health issue have focussed on the ubiquity of chronic pain and the fact of vastly unequal access to effective treatment, in particular access to opioids. In general, it is true to say that wealthier countries in the Global North have better access to opioids and other pain control technologies than do countries in the Global South [31]. In September 2015, the International Association for the Study of Pain promulgated the Declaration of Montréal, forcefully arguing that access to care (including opioids), de-stigmatisation of pain, better training of health professionals, and improved policies on pain management are essential for the improvement of pain worldwide. Indeed, the declaration is headed: ‘Declaration that access to pain management is a fundamental human right’ [32]. What is striking about the Declaration and similar formulations concerning questions about pain and human rights [33] is that the question of the prevention of chronic pain itself (rather than its medical management) is not considered. R’s story is one amongst many which amply demonstrate that adverse social conditions create an environment highly conducive to co-morbidity between pain and chronic stress, and hence to the development and maintenance of chronic pain.

The World Health Organization (WHO) Commission on the Social Determinants of Health report [34] makes it clear that, as Goldberg and McGee [30] put it, ‘remedies for the most pressing and inequitable global health problems are typically to be found outside the provision of health care services’ (para. 11). The WHO Commission report mentions prevention extensively (including prevention of injury and disease), but it does not explicitly discuss pain (in fact, the word ‘pain’ does not appear in the report). The report also does not mention PTSD and mentions trauma only once directly (on p. 98) in relation to the consequences of natural disasters, not in relation to social inequalities or what Nixon [35] refers to as ‘slow violence’ – the chronic consequences of inequality. The report outlines Three Principles of Action to address the social determinants of health:

1. Improve the conditions of daily life – the circumstances in which people are born, grow, live, work, and age.
2. Tackle the inequitable distribution of power, money, and resources – the structural drivers of those conditions of daily life – globally, nationally, and locally.
3. Measure the problem, evaluate action, expand the knowledge base, develop a workforce that is trained in the social determinants of health, and raise public awareness about the social determinants of health [34] (p. 2).

Interestingly enough, when the Commission’s Social Determinants of Health report is brought into the pain literature by Goldberg and McGee [30], though the spirit of these three principles can be detected subliminally in the text, the question of social action to attempt the prevention of chronic pain – and the researching of the effects, if any, of this social action – is not mentioned. So, in the WHO’s Social Determinants of Health report [34], chronic pain is not mentioned, and when the principles of this report are introduced to the chronic pain literature, engagement with the social conditions which may contribute to chronic pain is not mentioned.

## Conclusions

### An agenda for action

Chronic pain is well established as a global public-health issue, and it is important that access to treatment of chronic pain is improved globally – it is important that the IASP has identified pain relief as a human right [32]. It is also the case that chronic pain is an extremely complex phenomenon, with an as yet not fully understood interaction of physiological, psychological, and social factors contributing to the condition. To be more specific, the question of the context within which chronic pain occurs is not well examined in the literature, and the current study demonstrates the importance of that context.

The case we have briefly presented here in order to illustrate the argument is unique, as is every case, but it has features in common with a multitude of stories of the experience of pain, violence, social oppression, and exclusion in the majority world. It is not new to argue that bodily experience and experience of the body politic are enmeshed and intertwined, as medical anthropologists have been arguing for many years [36]. However, it is noteworthy that although chronic pain has been recognised as a human-rights issue, there is as yet no serious engagement with exploring the question of how improving human rights and social conditions may not only improve access to treatments for chronic pain but may also decrease rates of chronic pain. In this presentation, it has been suggested that a useful way to think about the links between chronic pain and human-rights issues is by reference to chronic stress in violent societies and communities. It is acknowledged, however, that at this stage, the ideas on this issue, though strongly clinically informed, remain at the level of theory. It is suggested that for researchers to take up the challenge of chronic pain as a human-rights issue fully, at least two forms of action need to be taken. First, research is urgently needed on the relationship between adverse social conditions and chronic pain and on the challenging question as to whether changing aspects of these conditions may change the development and experience of chronic pain. Second, more work at the interface between pain research (which has been dominated by questions of pain relief and management) and trauma studies (which has been dominated by psychological and psychiatric questions rather than by questions of embodied trauma) is urgently needed. This is required both so that chronic pain and its unequal distribution through the world is better understood and so that programmes and interventions with a focus on prevention of chronic pain can be developed, implemented, and evaluated. This will not solve the global problem of chronic pain by any means, but it may go some way to reducing the burden.

## Paper context

The relationship between chronic pain and PTSD is well established. There have also been critiques of PTSD as a concept from high-conflict and unequal societies. Continuous traumatic stress disorder may be a better concept for understanding reactions to ongoing violence and oppression. We present a case study to suggest the potential for a research agenda linking chronic pain and continuous stress as human-rights issues in such contexts.
